# Improving Treatment Adherence in Youths With Multidrug‐Resistant Tuberculosis With Psychosocial Intervention

**DOI:** 10.1002/brb3.70665

**Published:** 2025-08-04

**Authors:** Gauri Dhumal, Neetal Nevrekar, Nikhil Gupte, Rechel Shrisunder, Prashant Kulkarni, Sayali Sarpotdar, Akhil PM, Abhijit Hosmani, Rahul Lokhande, Amita Gupta, Nishi Suryavanshi

**Affiliations:** ^1^ Johns Hopkins Center for Infectious Diseases in India Pune Maharashtra India; ^2^ Byramjee Jeejeebhoy Government Medical College Clinical Trial Unit‐Johns Hopkins University Pune Maharashtra India; ^3^ Aundh Chest Hospital Pune India; ^4^ Byramjee Jeejeebhoy Government Medical College and Sassoon General Hospitals Pune India; ^5^ Johns Hopkins University Baltimore USA

**Keywords:** adolescents and young adults, intervention, MDR‐TB, psychosocial challenges

## Abstract

**Introduction:**

Multidrug‐resistant tuberculosis (MDR‐TB) deeply impacts the well‐being of adolescents and young adults (AYA), resulting in poor treatment adherence. Identifying psychosocial challenges and preferred interventions is essential to enhance treatment adherence and outcomes in this unique group.

**Methods:**

This was a mixed‐method study where participants aged between 15 and 24 years, diagnosed with MDR‐TB, were recruited for in‐depth interviews and a semi‐structured questionnaire.

**Results:**

The individual‐level psychosocial challenges included mental stress, suicidal ideation, reluctance to continue medication, perceived and experienced stigma, and socio‐economic burdens. Health system‐related challenges encompassed delayed diagnosis, drug stockouts, and negative experiences with Health Care Providers (HCPs). Among 75 participants, the median age was 20.5 years, with 57% (*n* = 41) females, 85% (*n* = 62) single, and a median treatment duration of 8 months at the interview. Seventy‐two percent (*n* = 54) of the participants reported psychological issues such as irritation, loneliness, anxiety, sleep disorder, suicidal ideation, and stigma. Individual‐level interventions were preferred by 61% (*n* = 46) of participants, including social media, deep breathing, and exercise training.

**Conclusions:**

To enhance results in MDR‐TB, it is crucial to develop and assess personalized psychosocial interventions with tailored adjustments to tackle the psychosocial obstacles encountered by adolescents and young adults with MDR‐TB.

## Graphical abstract

This study found that it is crucial to screen AYA with MDR‐TB for mental and social issues at the time of MDR‐TB diagnosis for early identification and intervention. There is an urgent need to include psychosocial intervention for AYA in TB program to improve programmatic treatment outcomes.

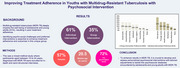

## 1 Introduction

Adolescence and young adults (AYA) are increasingly recognized as crucial periods for development (Sawyer et al. 2018; Patton et al. 2016) and a key risk period for tuberculosis infection, disease, and adverse outcomes (Marais et al. 2004; Morabia 2014). India has the largest AYA population, with 27.3% of the age group between 15 and 29 years, which is higher than any other country (National Statistical Office 2022).

Multi‐drug‐resistant tuberculosis (MDR‐TB) has emerged as a significant public health crisis in the 21st century (India TB Report 2023). Globally, between 2018 and 2019, there was a slight increase in the incidence and proportion of children and young adolescents receiving treatment for multidrug‐resistant (MDR) and rifampicin‐resistant tuberculosis (RR‐TB). RR‐TB refers to resistance to rifampicin, the most potent TB drug. However, treatment coverage remained low. India contributes the largest share (27%) of the global burden of MDR/RR‐TB, with an estimated 450,000 people affected in 2022 (World Health Organization 2023). Despite the availability of the medications, the current cure rate for MDR‐TB is 50% to 63% due to the long duration of treatment and non‐adherence to the drugs (World Health Organization 2023; Pradipta et al. 2018).

AYA with MDR‐TB faces specific age‐related challenges in accessing appropriate care (Sawyer et al. 2018). The period of this age group is marked by significant growth, hormonal changes, and development in various domains of life, as individuals prepare for the challenges and responsibilities of adulthood. It is a critical phase for building the physical, cognitive, emotional, and social resources needed for a successful transition into adulthood (Sawyer et al. 2018; Patton et al. 2016; Thakur et al. 2021). TB illness, especially MDR‐TB and its treatment, can intersect with the transitions and challenges that AYA faces during this critical period of development (Moscibrodzki et al. 2021). This will have an impact on how AYA experiences MDR‐TB illness and its treatment.

Few studies have identified adverse impacts of MDR‐TB treatment in adolescents, such as social isolation, stigmatization, depression, poor self‐esteem, and skin discoloration (Franck et al. 2014; Achar et al. 2015; Maymone et al. 2017). These create psychological distress for adolescents, leading to treatment discontinuation (Franck et al. 2014; Achar et al. 2015; Maymone et al. 2017). Two studies from South Africa and India reported delay in diagnosis, loss of follow‐up, and poor medication adherence as serious issues (Moyo et al. 2014; Isaakidis et al. 2013).

Adherence‐promoting interventions for adolescents are well‐established for HIV, diabetes, and other chronic diseases. However, there is a lack of evidence for tuberculosis‐specific adolescent‐friendly adherence support interventions. Few interventions that involved elements of directly observed therapy (Berrien et al. 2004; Gaur et al. 2010), pill swallowing (Garvie et al. 2007), and text message reminders (Dowshen et al. 2012) have not shown much success in retaining these youths in TB care. However, a systematic review showed that interventions based on psychosocial support, providing education, material support, and skill building that were incorporated into the standard of care, had a good impact on adherence and retention in care for PLHIV and TB. (Lyon et al. 2003; Law et al. 2019)

Addressing the psychosocial needs of AYA with MDR‐TB by identifying youth‐friendly psychosocial support interventions could improve adherence and treatment outcomes. Building on this, the present study seeks to document the psychosocial challenges faced by AYA with MDR‐TB and to establish an evidence base for identifying interventions that are acceptable to this population.

## 2 Methodology

### 2.1 Study Design and Participants

The study employed a sequential design, beginning with a qualitative phase followed by a quantitative phase. Six in‐depth interviews (IDIs) were conducted with participants in the age group of 18–24 years. They were selected to represent a range of ages (18–24), genders, and treatment durations. While thematic saturation was the aim, the limited number of interviews (*n* = 6) may not have fully achieved this. Quantitative participants (*n* = 75) were recruited using convenience sampling from the same hospital over 8 months. The sample size was based on feasibility rather than statistical power, and thus, the findings are exploratory. The interviews were conducted from April to October 2022 to understand the psychosocial challenges faced by AYA with MDR‐TB, their needs, and the motivators needed to retain them in care. They were recruited from the District TB Hospital, Pune, India. The IDIs were conducted for contextual understanding of psychosocial challenges and coping mechanisms adopted by AYAs.

During the quantitative phase, semi‐structured interviews were conducted with 75 participants (58 young adults and 17 adolescents), who were recruited from the District TB Hospital in Pune between January and August 2023.

### 2.2 Data Collection Procedure

Following the qualitative interviews, a focused literature review was conducted to identify evidence‐based psychosocial interventions used for adolescents and young adults with chronic illnesses, including TB. These included social media‐based awareness, deep breathing techniques, peer support, etc. The list was translated into the local language and explained in simple terms during the semi‐structured interviews, using culturally appropriate examples and visual aids when necessary. Following informed consent, trained social scientists conducted face‐to‐face interviews in the local language, maintaining the privacy of the study participants. These interviews aimed to identify psychosocial challenges encountered during the treatment journey and understand the utilization of any services and coping strategies adopted to address those challenges. All the interviews were audio‐recorded. No personal identifiers were revealed in the recorded transcripts. The duration of the interview was on average 45 min to 1 h.

### 2.3 Data Analysis

We used an inductive thematic approach. Initial codes were developed based on emergent patterns in the transcripts. Coding was performed independently by two researchers and then reconciled through team discussion to agree on the final code set. We utilized the DSM‐5 scale (American Psychiatric Association 2013) to measure the psychological challenges faced by AYA with MDR‐TB. The adult version (18 years and above) of the measure consists of 23 questions that assess 13 psychiatric domains, and the child‐rated version (11‐17 years) of the measure which we have used for our adolescent age group (15–17 years) consists of 25 questions that assess 12 psychiatric domains. Each item enquires how much (or how often) the individual has been bothered by the specific symptom during the past 2 weeks. Each item on the measure is rated on a 5‐point scale. For this study, we assessed seven domains, which focused on depressive symptoms; symptoms of anxiety, anger, somatic symptoms, suicidal ideation, sleep problems, and substance use, as these were the challenges reported by the participants during the qualitative phase of the study.

### 2.4 Statistical Analysis

#### 2.4.1 Qualitative

Audio recordings of the IDIs were transcribed verbatim in the local language (Marathi or Hindi) and translated into English. An experienced social scientist and the study co‐investigators agreed on a final code set for analysis.

Data were analyzed using structured thematic analysis by MAXQDA Analytics Pro 2022 software.

#### 2.4.2 Quantitative

Descriptive analysis was done for the semi‐structured questionnaire to understand the intervention preference with study variables by STATA Version 12.

#### 2.4.3 Ethical Approval

The study protocol was approved by the BJGMC ethics committee, India, and the Johns Hopkins University Institutional Review Board (JHU IRB), USA. Written informed consent was obtained from all participants. Audio recordings were transcribed verbatim, and member checking was done to ensure validity. Ethics approvals were secured, and informed consent was obtained from all participants. For adolescents (15–17 years), assent and parental consent were both obtained.

## 3 Results

### 3.1 Qualitative Results

For the qualitative assessment, six IDIs (4 females, and 2 males) were conducted with AYA with MDR‐TB with a median age of 21.5 years (IQR 18–24 years).

Table 1 highlights the two major broader areas in the interviews. Those were (1) psychosocial challenges faced at diagnosis of MDR‐TB disease and during treatment, and (2) coping mechanisms adopted by AYAs to mitigate these challenges. The psychosocial challenges were further categorized into sub‐themes; anticipated stigma, financial stress, career compromises along with the challenges to attend the school/colleges, emotional burden, fear of transmission of disease, high pill burden with adverse drug reactions, delay in diagnosis, and behavior of TB health care providers (HCPs). In addition, we found that AYA experience multiple compounding challenges in their MDR‐TB treatment journey—including stigma, economic strain, emotional burden, and healthcare system issues, all of which may negatively affect adherence and outcomes. AYA showed clear preferences for digital, individualized, and peer‐based support interventions. Coping strategies were categorized into sub‐themes, support from family, friends, and HCPs, and other motivational drives.

**TABLE 1:** Psychosocial challenges and coping strategies of AYA with MDR‐TB in India.

Themes	Interpretation	Quotes
Psychosocial Challenges		
Anticipated stigma	The interviewed respondents expressed reluctance to disclose MDR‐TB status to family and friends, distancing themselves socially during treatment. One female respondent experienced isolation from friends and sadness due to physical side effects, such as red rashes, impacting family gatherings and social functions.	“*If they (friends/society people) come to know (about the disease condition) they would go away so people do this (not to disclose). There are such friends if they come to know their mother tells them that this disease spreads, so don't be in his/her contact”. (Female, 18 years)*
Financial stress	The respondents had to face financial hardships due to limited earning members and the income or due to loss of job because of the disease condition.	*"They were saying it needs free air and it is all congested where we stay. Our house is at the end. We have searched for another house, but money is required for rent and deposit. My maternal aunt's son looks after us. My father's salary is less, and my mother and I have to eat fruits and eggs every day. So, we are not leaving this place”. (Female, 18 years)*
Career compromises	The longer treatment period of MDR‐TB and the pill burden often adversely impact the career pursuit among young patients and can potentially lead to emotional distress.	*“Because of this infection (MDR‐TB disease), my dream of going into defence also remained incomplete.” (Male, 22 years)* *“I did not go to school. I left my schooling after that (after being diagnosed with MDR‐TB disease)” (Female, 23 years)*
Emotional burden	One respondent revealed a household history of MDR‐TB transmission, expressing apprehension about negative treatment outcomes. Respondents commonly experienced stress and tension related to treatment, particularly during discussions with HCPs regarding missed doses. In addition, they reported feeling stressed upon initial diagnosis of MDR‐TB.	“*There were previous two deaths at my home, both of my brothers succumbed to MDR‐TB. My mother got MDR‐TB and has been admitted in the hospital for the past 26 days”. Father is the only earning member. (Female, 18 years)* *“Yes, sometimes they (HCP) used to shout when I used to go for taking medicines when I used to be late for 1 to 2 days for taking the medicines, they (HCPs) used to say that we will not give medicines again”. When they used to shout at that time, I used to get scared. They (HCPs) said “Why didn't you come (on time) to take the medicines? At that time, I used to feel scared” (Female, 23 years)*
Fear of transmission	Respondents voiced apprehension regarding disease transmission, despite constrained living spaces prompting cohabitation. Two of six respondents noted unfavorable living conditions, including overcrowding and insufficient ventilation. One respondent specifically described four individuals occupying in one small room measuring around 10*10 square feet.	“*At home two brothers, mom‐dad, sister, she is married but she stays with us only. She has two small kids; the kids are small. Our home is small, one room is there” (Female, 23 years)*.
High pill burden correlates adverse drug outcomes	Respondents reported a substantial pill burden, averaging at least 13 tablets daily, contributing to increased discomfort. Side effects from anti‐TB drugs were universally experienced, with common complaints including post‐medication vomiting, nausea, and breathlessness during physical activity. In addition, non‐adherence to medication was observed to impact mental health in some respondents negatively.	“*I used to feel like quitting medicines sometimes, one day I was so burdened (kantala yene) (of taking medicines), like I felt I don't want these tablets, and I used to get irritated”. (Female, 23 years)* *“Injections were taken every day on one side (gluteal region). Once on this side, once on that. My thighs used to ache. If I walked more, my knees would also ache. I wasn't able to walk for long*.*” (Female, 24 years)*
Delay in diagnosis, and behavior of HCPs	Respondents commonly exhibited prolonged symptoms attributable to delayed diagnosis. Notably, all individuals sought initial medical consultation from private practitioners upon symptom manifestation.	*“Fever was there for 3 months continuously, then after doing checkup at Aundh Hospital (A hospital in Pune) it was diagnosed as MDR‐TB”. (Male, 22 years)*
**Coping strategies**		
Support from family, friends and HCPs	The respondents mentioned that support from family, friends, and HCPs is very important for a fast recovery.	*“I tell everything to my friend. She says that I will be alright soon so I can go out. She asks me to take medicines, eat properly, and gain weight (jaadi‐moti ho ja). Your illness will be recovered, and you will be fine. Then you can go to all the places” (Female, 18 years)* *“They (HCPs) used to talk well because of which I used to feel courageous.” (Female, 23 years)*
Other motivational drives	Respondents used drawing, listening to motivational songs, and trekking as their coping strategies during the phase of treatment. Respondents said that they have consoled themselves. Respondents adopted strategies like chanting God's name. Two of the respondents used a diary as a means for venting their feelings.	*“I used to follow God, used to read Hymn (Adhyay)”. (Female, 24 years)* *“In mobile, I used to watch motivational videos more about MDR‐TB, did not watch demotivational videos”. I have a diary. I used to write that in treatment till where I have reached. I used to write poetry. (Male, 19 years)*

### 3.2 Quantitative Results

Table 2 highlights the association of socio‐demographic factors with the type of intervention preferred by the semi‐structured survey participants. Of 75 participants, with a median age of 20 years (IQR 18–22 years), 57% (*n* = 41) were female, 76% (*n* = 55) belonged to the Hindu community and 86% (*n* = 62) were single. Over 61% (*n* = 44) of them had completed more than 10 years of education, 26% (*n* = 19) were employed, 49% (*n* = 35) were students, 61% (*n* = 44) belonged to the upper‐lower socioeconomic class, 83% (*n* = 60) were from the nuclear family, 79% (*n* = 57) had own mobile phone and 96% (*n* = 55) had internet facility.

**TABLE 2:** Association of socio‐demographic factors with the type of intervention preferred.

Characteristics		Overall	1. Individual Interventions, *N* = 46^1^	2. Group Interventions, *N* = 26^1^	*p*‐value (**Patton et al**. 2016)
**Age**	15–17	16 (22%)	10 (22%)	6 (23%)	0.4
	18–21	35 (49%)	25 (54%)	10 (38%)	
	22–24	21 (29%)	11 (24%)	10 (38%)	
**Gender**	Male	31 (43%)	20 (43%)	11 (42%)	>0.9
	Female	41 (57%)	26 (57%)	15 (58%)	
**Education**	≤10 years	28 (39%)	21 (46%)	7 (27%)	0.12
	>10 years	44 (61%)	25 (54%)	19 (73%)	
**Employment status**	Employed	19 (26%)	8 (17%)	11 (42%)	0.032^*^
	Student	35 (49%)	23 (50%)	12 (46%)	
	Unemployed	18 (25%)	15 (33%)	3 (12%)	
**Employment categories**	Unemployed	18 (48%)	15 (65%)	3 (21%)	0.006^*^
	Unskilled worker	2 (0.5%)	1 (4.3%)	1 (7.1%)	
	Skilled or semi‐skilled worker	10 (27%)	2 (8.7%)	8 (57%)	
	Professional or semi‐professional worker	5 (13%)	3 (13%)	2 (14%)	
	Clerical/Shop/Farmer	2 (0.5%)	2 (8.7%)	0 (0%)	

* Statistically significant.

### 3.3 Psychosocial and Other Challenges Faced by AYA With MDR‐TB

**FIGURE 1:**
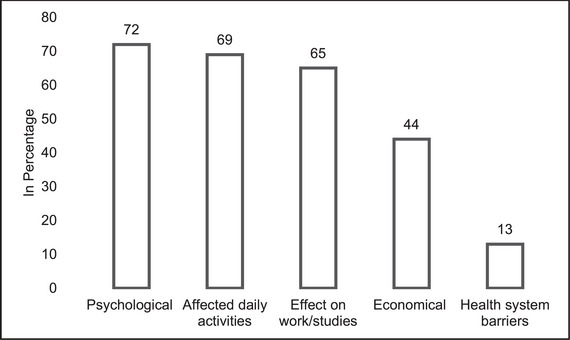
Challenges faced during the treatment. **Psychological challenges** include anger/irritation, stress, sleep problems and low feeling; **Affected daily activities** include disturbances in the sleep‐wake cycle and challenges in managing routine activities; **Effects on work/studies** include unable to concentrate on work, memory issues, lost self‐desire to study, scored low grades, school/college dropouts; **Economic challenges** include challenging to meet living and the travel cost. **Health system‐related challenges** include delay in diagnosis and delay in treatment initiation.

Figure 1 presents common challenges reported by AYA with MDR‐TB. One‐third of the population (72%; *n* = 54) documented psychological challenges where they mentioned anger/irritation (57%, *n* = 43), stress (54.6%; *n* = 41), inability to sleep (54.6%; *n* = 41), and feeling low (53%; *n* = 40). Social challenges reported were effects on work/studies (65%; *n* = 49), where they mentioned they were unable to concentrate on work (53%; *n* = 40), had memory issues (33%; *n* = 25), lost self‐desire to study (40%; *n* = 27), scored low grades (23%; *n* = 8), school/college dropouts (30%; *n* = 12). Economic challenges were reported by 44% (*n* = 48) of the participants where they mentioned it was challenging to meet living (65%; *n* = 49) and travel costs (48%; *n* = 36).

In addition, a notable number of participants (69%; *n* = 52) experienced impacts on daily functioning including disturbances in the sleep‐wake cycle (63%; *n* = 47) and challenges in managing routine activities (69%; *n* = 52). Health system‐related challenges included delay in diagnosis (21%; *n* = 16) and delay in treatment initiation (20%; *n* = 15).

Psychological challenges enquired by using the DSM‐5 scale are highlighted in Table 3 where 39% (*n* = 29) of AYA reported somatic symptoms, 40% (*n* = 30) reported symptoms of anxiety, 36% (*n* = 27) had depressive symptoms, 39% (*n* = 29) had anger, 31% (*n* = 23) had sleep problems, with few substances use and suicidal ideation.

**TABLE 3:** Psychological challenges documented by DSM‐5 scale^*^.

Sr. no.	Domain names	Adult and adolescent (*n* = 75)	Reference range
1	Somatic symptoms	29 (39%)	≥2 (Mild or greater)
2	Symptoms of anxiety	30 (40%)	≥2 (Mild or greater)
3	Symptoms of depression	27 (36%)	≥2 (Mild or greater)
4	Anger	29 (39%)	≥2 (Mild or greater)
5	Sleep problems	23 (31%)	≥2 (Mild or greater)
6	Substance use	3 (4%)	≥1 (Slight or greater)
7	Suicidal ideation	3 (4%)	≥1 (Slight or greater)
8	Inattention	3 (4%)	≥1 (Slight or greater)

* DSM‐5 Scale (American Psychiatric Association 2013): The Diagnostic and Statistical Manual of Mental Disorders Scale.

### 3.4 Preference for Psychosocial Interventions

**FIGURE 2:**
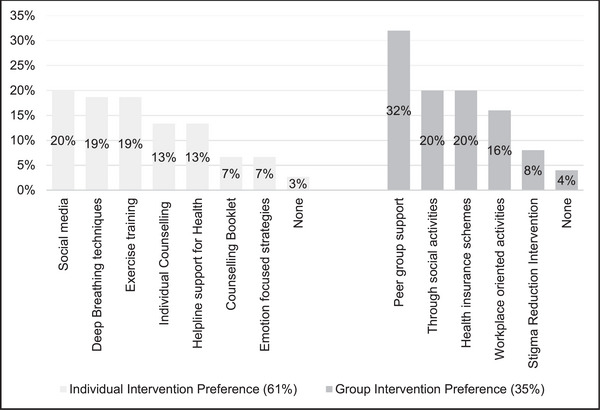
Psychosocial intervention preferences. **Individual interventions include** social media, deep breathing techniques, exercise training, Individual counseling, helpline support for health, counseling booklet, emotion‐focused strategies; **Group interventions include** peer group support, Through social activities, Health insurance schemes, Workplace oriented activites, stigma reduction intervention.

Overall, 61% (*n* = 46) of participants expressed a preference for individual interventions, 35% (*n* = 26) preferred group interventions, and 4% (*n* = 3) indicated no particular preference (Figure 2).

### 3.5 Preferred Individual Intervention

Audio‐visual aids represent a form of individual intervention; however, many participants unwittingly favored these aids as coping strategies, which highly motivated them to adhere to treatment and seek mental health support. Participants reported a preference for social media/video education awareness (20%; *n* = 15) followed by deep breathing/relaxation techniques (19%; *n* = 14), and exercise training/physiotherapy (19%; *n* = 14) under individual intervention (**Figure** **2**). The reasons provided for preferring this intervention through social media were availability, literacy, and its extensive use among this population as quoted below.

“Nowadays, people's attention has been shifted to OTT platforms. OTT platforms stand for ‘Over‐The‐Top’. It refers to streaming services that deliver video content over the internet directly to users. So, people will surely go for it. And as we know, in India, there are a large number of youths, and they mostly make use of social media.” (Male, 16 years)

“It will be useful for youths. As we mostly make use of social media and other related things. We will surely pay attention to whatever is shown related to disease.” (Female, 15 years)

### 3.6 Preferred Group Intervention

Peer group support/knowledge sharing programs (32%; *n* = 24) followed by an awareness drive through social programs/through celebrity voice (20%; *n* = 15), and extensions of health insurance schemes (20%; *n* = 15) were the first 3 choices reported as group interventions by the participants. These group interventions were preferred due to learning from the experience of other peers and seeking mental and emotional support during the treatment journey.

“It can help us. Other people will benefit from their experiences. We can also be aware of someone if they have had bad experiences regarding treatment. These are the people who can accurately share their views about it. (So, the group intervention will be) Most beneficial.” (Male, 24 years)

“We can relate to their experiences and can easily understand whatever they are talking about. I will ask about their routine, how they (peers) proceeded with self‐management and medicines etc.” (Male, 16 years)

## 4 Discussion

To the best of our knowledge, this is the first study in India to understand the psychosocial challenges and preferable interventions for AYAs to address those during their MDR‐TB treatment to retain them in care. However, other studies aiming at psychosocial challenges were focused on other disease conditions such as HIV/AIDS, leprosy, and other chronic conditions. (Govindharaj et al. 2016; Borah et al. 2018)

AYA with MDR‐TB in the present study reported social isolation, socio‐economic burden, anticipated stigma, mental stress, depression, poor self‐esteem, and career compromises as major psychosocial challenges faced by AYA which had also been reported in other studies (Walker et al. 2017). A systematic review by Sutar et al. (2024) underscores the significant prevalence of anxiety and stress in TB patients, emphasizing the urgent need to integrate mental health services with TB care, especially in low‐ and middle‐income countries. Our study echoes this need among AYA with MDR‐TB, aligning with findings in adult populations (Sutar et al. 2024). This highlights the need to screen this vulnerable population for their mental health status at the time of MDR‐TB diagnosis and provide support throughout the MDR‐TB treatment phase. In addition, counseling support is important while initiating the treatment.

To overcome these challenges, individual‐level interventions utilizing social media, such as OTT platforms to generate content on mental health intervention, and one‐to‐one counseling were reported as the most preferred interventions by AYA. Anticipated stigma, mistrust in healthcare systems, lack of awareness, and reluctance to seek in‐person help for mental health issues reported among adolescents lead to the use of the Internet as an alternative resource for their help (Schueller et al. 2017; Sweeney et al. 2016). Similar to our study, the Internet/social media will help to find support when experiencing mental health challenges (Gould et al. 2002; George et al. 2021). Also, online services are reported to be more appropriate for issues around which there is a high proportion of perceived stigma (Walker et al. 2018). Our study findings concur with other studies about how providing health education, counseling, and peer support can be valuable and transformative components of psychosocial interventions, particularly for AYA with MDR‐TB (Walker et al. 2018; Maynard et al. 2023; Jauhar Fuadi et al. 2019). However, it is important to develop strategies focusing on increasing knowledge of online therapies among these populations and target the ones who are already engaged in digital platforms (Walker et al. 2018). Sustainable models for mental health integration, such as consultation‐liaison psychiatry, are crucial to reduce psychosocial burdens in MDR‐TB care (Sutar et al. 2023). Strengthening systemic frameworks within TB programs can enhance patient outcomes and ensure continuity of mental health support. Our findings align with recent literature that highlights the need for tailored psychosocial interventions among youth with TB (Chiang, Senador, et al. 2023; Chiang, Waterous, et al. 2023).

Indian TB guidelines (2023) provide a facility for psychiatric referrals (India TB Report 2023). However, its implementation status is still not yet clear. Lack of enough trained psychiatrists and psychologists is a challenge for the program and hence utilizing social media and training peers to provide psychosocial counseling could be the best option to explore. TB healthcare system can leverage social media platforms along with digital intervention as well as peer counseling (Jauhar Fuadi et al. 2019) to mitigate psychosocial challenges faced by AYA with MDR‐TB and promote treatment outcomes and retention in care.

### 4.1 Strengths and Limitations

This study's strength is its ability to understand the psychosocial challenges faced by AYA during treatment and to identify the coping strategies they adopted to address these issues. It enables the establishment of predefined quantitative metrics for psychological distress and facilitates the documentation of preferred interventions across different disease conditions.

This study did not include a comparison group of AYA without TB or with drug‐sensitive TB, which limits our ability to distinguish which psychosocial burdens are specific to MDR‐TB. In addition, the study was not powered to analyze subgroup differences (e.g., by gender or age band), which warrants further exploration in future research.

The sample size is small due to the time constraint of the data collection. The data collection focus was restricted to the registered participants from only one district tuberculosis hospital. Along with that we have only captured the AYA's perspectives; however, understanding the pathways of infection, index cases within the family, and the family/care‐giver's perspective regarding the disease and treatment experiences would have contributed to getting a holistic understanding of the psychosocial challenges and the strategies to overcome those. Specifically, DSM‐5 mental health domains were considered for the study, however, not all domains were considered. The small sample size and single‐site recruitment limit the generalizability of the findings. In addition, the absence of stratified analysis by key subgroups (e.g., age, gender) restricts interpretation. Future research should use participatory, co‐designed methods and be adequately powered to investigate these dimensions.

We have looked at the patient's perspective on the intervention preferences. However, the HCP's perspectives about the mental health issues faced by AYAs and viable intervention options to address them have not been addressed.

## 5 Conclusion and Recommendation

Given the exploratory nature of our study, we recommend that the intervention model developed for AYA with HIV or adults with TB may be adapted for AYA with MDR‐TB. However, large, more diverse studies using a participatory model are needed to design evidence‐based culturally tailored psychosocial interventions.

We also recommend tailor‐made counseling based on the participants' psychosocial needs. Providing counseling on the drug regimen, pill burden, importance of treatment adherence, and side effects of the drug regimen during the treatment initiation would minimize the emotional distress among the AYAs. Prior information would make them aware and prepared to accept and adhere to the treatment. Using innovative audio‐visual‐based Information Education and Communication (IEC) materials in MDR‐TB treatment facilities can be a stepping stone for such counseling services and psychosocial interventions.

## Author Contributions

**Gauri Dhumal**: conceptualization, methodology, investigation, funding acquisition, writing – original draft, validation, supervision, project administration, writing – review and editing, visualization, resources, data curation, formal analysis, software. **Neetal Nevrekar**: conceptualization, investigation, methodology, validation, visualization, writing – review and editing, data curation. **Nikhil Gupte**: software, data curation, formal analysis, writing – review and editing, methodology. **Rechel Shrisunder**: investigation, validation, visualization, formal analysis, writing – review and editing, data curation, methodology, software. **Prashant Kulkarni**: data curation, formal analysis, investigation, methodology, validation, visualization, writing – review and editing, software. **Sayali Sarpotdar**: project administration, writing – review and editing, investigation. **Akhil PM**: software, data curation, formal analysis, supervision, validation, methodology, writing – review and editing. **Abhijit Hosmani**: resources, supervision, writing – review and editing, visualization, validation, methodology. **Rahul Lokhande**: methodology, validation, visualization, investigation, writing – review and editing, project administration, resources, supervision. **Amita Gupta**: methodology, validation, investigation, visualization, writing – review and editing, software, formal analysis, project administration, data curation, supervision, resources. **Nishi Suryavanshi**: conceptualization, investigation, funding acquisition, writing – original draft, methodology, validation, visualization, writing – review and editing, software, project administration, supervision, resources.

## Conflicts of Interest

The authors declare no conflicts of interest.

## Peer Review

The peer review history for this article is available at https://publons.com/publon/10.1002/brb3.70665

## Data Availability

Data will be available on request.
